# The impact of a community based rehabilitation program in Afghanistan: a longitudinal analysis using propensity score matching and difference in difference analysis

**DOI:** 10.1186/s13031-021-00397-y

**Published:** 2021-08-21

**Authors:** Jean-Francois Trani, Juanita Vasquez-Escallon, Parul Bakhshi

**Affiliations:** 1 Institute of Public Health, Brown School, Washington University in St Louis, Campus Box 1196, Goldfarb Hall, Room 243, One Brookings Drive, St. Louis, MO 63130 USA; 2 2UNICEF, Phnom Penh, Cambodia; 3 Institute of Public Health, Program in Occupational Therapy, Washington University School of Medicine, St. Louis, USA

**Keywords:** Afghanistan, Community based rehabilitation, Difference in difference, Disability, Impact evaluation, Low middle income countries, propensity score matching

## Abstract

**Background:**

The 2006 United Nations Convention on the Rights of Persons with Disabilities states that the achievement of equal rights, empowerment and social inclusion of people with disabilities requires comprehensive rehabilitation services encompassing all components of the World Health Organization Community based rehabilitation (CBR) matrix: health, education, livelihood, social and empowerment. CBR programs specifically aim to deliver such comprehensive interventions. In the present study, we investigate the impact of a CBR program in Afghanistan on all these components.

**Methods:**

We enrolled 1861 newly recruited CBR participants with disabilities in the study, from 169 villages between July 2012 and December 2013 as well as 1132 controls with disabilities randomly selected through a two-stage process within 6000 households from 100 villages in the same provinces but outside the catchment area of the CBR program. We interviewed them again after one (midline) and two (end-line) years in the study. Using propensity score matching and difference in difference analysis, we estimated the impact of the CBR on outcomes of interest, namely mobility, activities of daily living, communication, participation in social and community life, emotional well-being and employment.

**Results:**

Three years on average into the CBR program, participants showed a significant and close to medium effect size reduction in emotional (Cohen’s d = − 0.48, 95%CI[− 0.58--0.38]), and social participation challenges (Cohen’s d = − 0.45, 95%CI[− 0.53−− 0.36]); small to medium effect size reduction in unemployment (Cohen’s d = − 0.21, 95%CI[− 0.33--0.10]), activities of daily living (Cohen’s d = − 0.26, 95%CI[− 0.35--0.18]), mobility (Cohen’s d = − 0.36, 95%CI[− 0.44--.29]) and communication challenges (Cohen’s d = − 0.38, 95%CI[− 0.46--0.3]).

**Conclusions:**

Our study indicates that a CBR program may provide positive rehabilitation outcomes for persons with disabilities even in a conflict context, and improve overall well-being of all participants with disabilities, whatever their impairment, individual characteristics and the CBR matrix components considered.

**Trial registration:**

ISRCTN, ISRCTN50214054. Registered August 5th 2020 - retrospectively registered

## Background

The 2006 United Nations Convention on the Rights of Persons with Disabilities (UNCRPD) states that the achievement of equal rights, empowerment and social inclusion of people with disabilities requires comprehensive rehabilitation services encompassing educational, social, economic and medical interventions [1]. In particular, article 26 of the UNCRPD calls for rehabilitation services and programs to promote more participation of persons with disabilities in their community and in all aspects of broader society. Community Based Rehabilitation (CBR) is a strategy that promotes the “rehabilitation, equalization of opportunities, poverty reduction and social inclusion of all people with disabilities” [2, 3].

CBR programs were introduced in the 1970s as a participatory strategy to use effective, locally-developed technologies and interventions to prevent disability and transfer knowledge and skills about disability and rehabilitation to people with disabilities, their families and the community at large involved in the program. CBR was conceived as a combined effort of a diverse group of actors, including families, communities, disabled people’s organizations, health and social services provided by governmental and non-governmental actors, and, at the center, people with disabilities themselves.

Advocates of CBR identify several advantages of this approach over other alternatives. First, CBR is comprehensive; experts have demonstrated that all rehabilitation needs can be addressed through CBR interventions [4–6]. Second, authors have argued that CBR programs are more cost-effective than hospital or rehabilitation center-based interventions [7]. Third, taking a strong rights-based approach, CBR aims to specifically improve the wellbeing of systematically marginalized people with disabilities [8]. Finally, CBR is oriented toward participation and empowerment of people with disabilities [9, 10].

Despite the stated strengths, CBR programs continue to face several critiques linked to field realities. The first is that, while nominally based in values of participation and empowerment of people with disabilities, CBR often reproduce the same top-down service delivery approach of other methods [11]. Second, it has been argued that CBR are operated and funded by international aid and humanitarian organizations, raising significant questions about the sustainability of programs when donor priorities change [11]. Finally, at implementation level, many programs have scarce resources and lack strong support from the community. Absence of community involvement leads to poor monitoring despite some recent progress with the elaboration in partnership with CBR stakeholders of a monitoring manual [12–14]. It also results in limited ownership, empowerment and program relevance and sustainability [8, 15].

Despite the proposed benefits of CBR, extensive empirical literature that provides evaluation of the impact of CBR programs in diverse contexts is lacking. Most existing studies do not evaluate the overall WHO CBR matrix but overwhelmingly focus on its health component [15, 16].

Moreover, studies often focus on one condition or type of disability and do not evaluate the impact of CBR programs across disabilities. Notwithstanding the focus of the CBR matrix and the program design on the participation of people with disabilities in communities, few studies examine the contribution of CBR towards fostering empowerment and increasing social inclusion of people with disabilities and their families or change in community attitudes and behavior towards people with disabilities [8, 13, 17]. The general lack of overall evaluation studies is also explained by the absence of standardized outcomes and of a discrete intervention [18].

Finally, sustainability of CBR is overall neglected [19]. This research gap is in part the consequence of the prioritization of implementation over evaluation in CBR by development organizations, funders, and policymakers. Most existing research focuses on accessibility, reach of the program, identification of needs and specific rehabilitation and service delivery outcomes [20]. Studies that do exist lack consistent methodologies, making comparison across programs challenging and unreliable [15, 21–23]. For instance, existing CBR program evaluation studies have non-experimental design with limited size samples [15].

The present impact evaluation study carried out between July 2012 and December 2015 contributes towards filling the gap between theoretical concepts of CBR and the actual completion of a CBR program implemented in 13 provinces of north and eastern Afghanistan by measuring the impact of CBR activities on the circumstances and well-being of participants with disabilities. The study aims at advancing knowledge on CBR program effectiveness by investigating the effect of the Swedish Committee for Afghanistan CBR program on mobility, activities of daily living, communication, social participation, emotional well-being and access to employment of persons with disabilities.

## Methods

### Study aim, design and setting

#### Aim and design

The present study investigates the impact of the Swedish Committee for Afghanistan (SCA) CBR using a quasi-experimental design on major outcomes of interest after a maximum of 36 months in the study: How do persons with disabilities who received the CBR program’s services fare in terms of challenges in mobility, activities of daily living, communication, social participation, emotional well-being and employment compared to control with disabilities?

We used a quasi-experiment because we could not randomly select and assign treatment and control to the two arms of the experiment. The program is offered to all persons with disabilities living in its catchment area and it would have been both unpractical and unethical to withdraw the program to some potential participants. We used propensity score matching (PSM) associated with difference in difference (DD) analysis to minimize critical differences between the treatment and control groups and yield unbiased robust estimates of program effects using a non-parametric regression [24]. PSM was used to balance baseline characteristics between treatment and control groups in order to construct an analytical sample in which treatment and control groups are exchangeable [25]. DD analyses adds to PSM by further disentangling the program effect from differences between treatment and controls and from unrelated time-based trends in the outcome. DD allows a longitudinal analysis at two times points (at baseline and again after a maximum of 36 months after baseline) of a difference in outcomes (i.e. mobility, activities of daily living, communication, social participation, emotional well-being and employment) considering a difference in participation in the CBR program [26].

#### Settings

**Fig. 1:**
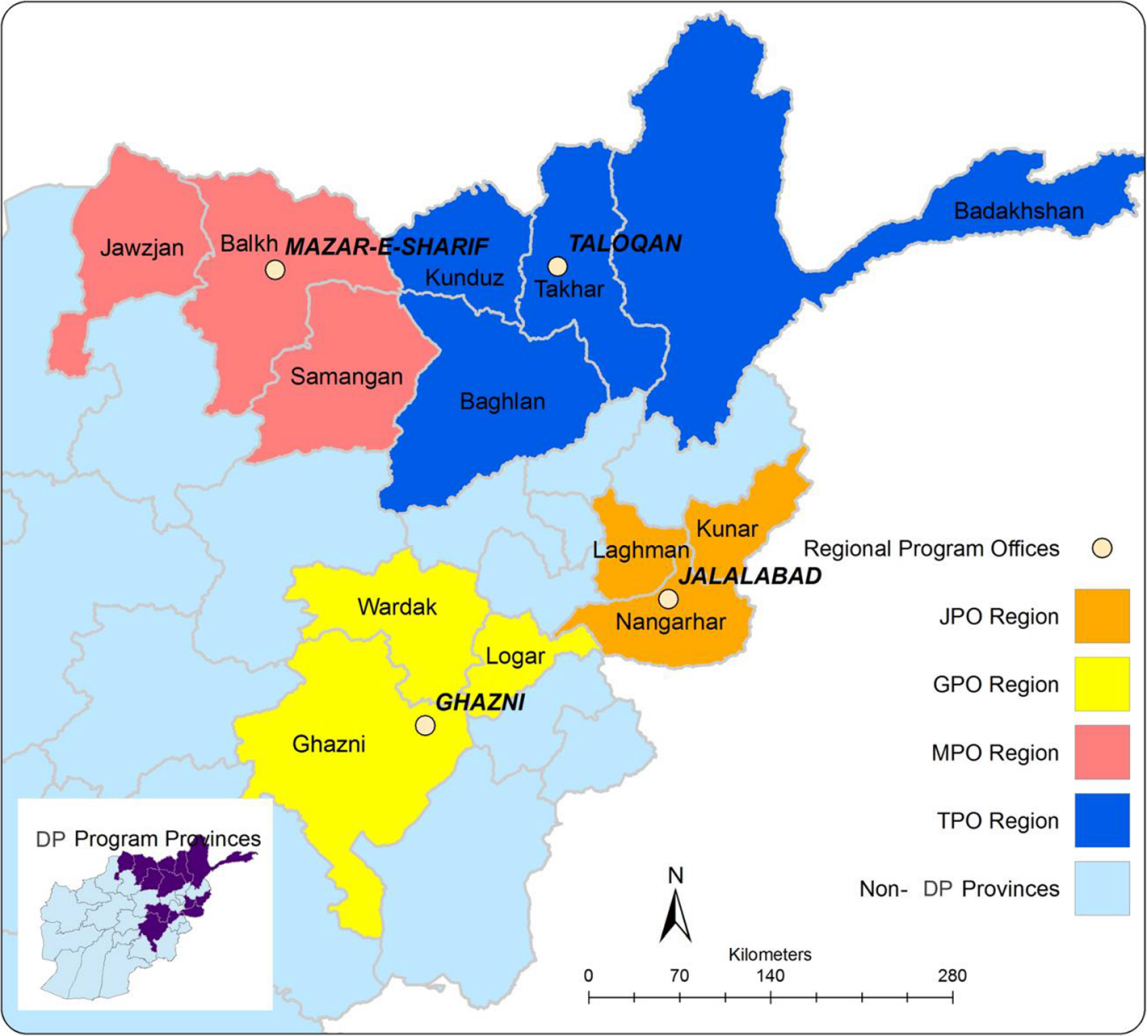
Map of the CBR program interventions areas. JPO: Jalalabad Provincial Office, GPO: Ghazni Provincial Office, MPO: Mazar-I-Sharif Provincial Office, TPO: Taloqan Provincial Office

The CBR is implemented in 13 provinces of northern and eastern Afghanistan (See Map 1) and provides services to an estimated 2301 persons with disabilities in home-based activities and 1443 children in home-based education in 2018. The program covered 48 districts with over 775 staff, 863 (413 female) community volunteers and 151 (60 composed of female) CBR committees. The program is managed from four regional project offices based in Ghazni city (Ghazni province), Jalalabad (Nangharar province), Mazar-e-Sharif (Balkh province) and Taloqan (Takhar province) (See Fig. 1).

### Participant characteristics

#### Selection of program participants

For the present study, we interviewed all new 1680 CBR participants included in the program between July 2012 and December 2013. Besides living in one of the 169 villages or urban areas (*mahals)* of the catchment area of the program, the other inclusion criteria were the ones defined by the CBR program at its start in 2004. At the creation of the program, inclusion was defined under the following criteria: (i) having a disability defined by the WHO guidelines for grassroot disability programs to account for contextual factors as having difficulties (e.g. seeing, hearing, speaking, learning or moving around, behavioral difficulty) that make it challenging to conduct all the activities that other members of the family or the community do, resulting in community members often considering people with such difficulties as being different or inferior to others and treating them unfairly [27]. Persons were screened for disability using a locally developed, tested and validated questionnaire based on these WHO guidelines; (ii) living in the areas or *mahal* close to the other SCA activities such as orthopedic workshops and physiotherapy centers/clinics; (iii) *mahals* had to be the place of residence of the newly recruited CBR workers before the program could expand progressively to nearby villages until it covered the entire district; iv) the absence in the *mahal* of a similar intervention by any other organization, and v) the intervention had to be welcomed by the overall village community and particularly the village council or *shura;* vi) the willingness of person with disabilities and the family members to participate in the CBR programs; and vii) the readiness of a family member to be trained by —and implement— the activities set up by the CBR worker in order for such activities to be ongoing daily, while the CBR worker would check progress made on a weekly or sometimes bi-weekly basis. If a village on the province was not part of the program for whatever reason, it was automatically included in the list of villages for random selection of controls. On average, one CBR worker was serving 100 participants with disabilities per year. Each expansion was decided in agreement with the CBR program management and the new targeted areas were surveyed for identification of persons with disabilities using the same WHO instrument [27].

#### Selection of controls

Controls were randomly selected during the same period with a two-level selection process: At first level, a random group of villages and urban areas of the same provinces but outside of the catchment area of the CBR program and at the second level a random group of households within each village or urban area (See Appendix for more details about the control group selection process).

### Study tools and data collection

#### Disability screening

All heads of households were interviewed with a locally validated disability-screening tool composed of 34 items for adults (DSQ-34) and 35 for children (DSQ-35) based on the same disability definition as participants to identify all members of the household with disabilities [28].

#### Questionnaire development and testing

All study participants were interviewed by the CBR workers with a locally developed and validated questionnaire that inquired about demographic characteristics, socioeconomic status, access to rehabilitation, health and social services, individual functioning, social participation, and additional needs. The questionnaire examined the effectiveness of the CBR program in improving the agency of persons with disabilities to determine their daily lives, participate in different aspects of community life, escape stigma and prejudice, and access various CBR services from the five domains of the CBR matrix (health, education, livelihood, social inclusion and empowerment) [2]. Disability experts in Afghanistan were asked to review the content of the initial English version of the tool for completeness, content validity, and appropriateness of the questions to the Afghan cultural context. The English version of the tool was then translated into Dari and Pashto by a disability expert from the Ministry of Public Health in Kabul. Several different translators worked independently to back-translate the survey into English and compare results to reconcile discrepancies. A first version of the questionnaire developed by the authors was initially tested end of 2011 with a group of 20 CBR participants in Jalalabad, Nangarhar, Afghanistan. Each respondent was interviewed separately by a researcher for consistency check of responses provided. Additionally, the Dari and Pashto versions of the final questionnaire were tested through a series of 30 interviews in Kabul in 2012 with persons with disabilities of different age groups, gender and ethnicity to verify that response process followed, understanding and interpretation of complex or technical terms, such as access to healthcare, available CBR services, participation in family and community activities, and measures of additional needs as well as satisfaction with life were consistent across different socioeconomic background and with the initial concepts conceived in English by the researchers. Respondents were asked the questions as defined by researchers followed by a series of probe questions aiming at capturing their understanding of the questions in light of their own life experience [29].

#### Data collection

All study participants were interviewed with the same tool three times respectively between July 2012 and December 2013, between July 2013 and December 2014 and finally between July 2014 and December 2015.

### Variables

#### Outcome variables

Six main outcomes of interest were included in the questionnaire and assessed through a range of questions: mobility, activities of daily living, communication, participation in social and community life, emotional well-being and employment. Indexes for each domain were created by generating a sum index score from the component items in the questionnaire. Because outcomes were sometimes different between age groups (for example, questions pertaining to ability to bathe oneself were not asked of infants too young to do so on their own), sum index scores were based on total points possible for each age group. Each summary index score was then divided by total points possible according to age and converted to a proportional value between 0 and 1. Difference scores between rounds 3 and 1 were then calculated. Thus, a result of .15 indicates a 15% increase in points possible within a given domain. Only employment did not result in an index.

#### Mobility index

The mobility index was composed of five activities with response choices limited to a Likert scale composed of three choices (I can always, I can with help, I cannot at all): Can you sit (asked to respondents above 1 year old); Can you stand (above 1 year old); Can you move inside the home (above 1 year old); Can you move outside the home (above 2 year old); Can you walk at least ten steps (above 2 years old).

#### Activity of daily living index

The Activity of daily living index was composed of four activities with the same three response choices: Are you able to eat on your own (asked above 4 years old); Are you able to bathe (above 8 years old); Are you able to use the latrine (above 3 years old); Can you dress and undress (above 4 years old).

#### Communication index

The communication index focused on the four following functions with the same three response choices: Can you speak (above 2 years old); Can you understand simple instructions (above 2 years old); Can you express needs (above 2 years old); Do you feel confident learning new things (above 4 years old).

#### Social participation index

The social participation index comprised a first item below with three response choices (I can without difficulty, I can with some difficulties, no, I cannot at all) and four following items with three different choices (I can always, I can sometimes, no never): Can you make friends outside the family; Are you consulted in family decisions (above 15 years old); Can you join in community activities and ceremonies; Do you feel respected in the community (above 5 years old); Do you feel respected in your family.

#### Emotional well-being index

The emotional well-being index was composed of five items with three response choices (never, sometimes, always): Do you feel sad (above 5 years old); Do you feel angry (above 5 years old); Do you feel worried or distressed (above 5 years old); Do you have nightmares or bad sleep (above 5 years old); Do you have headaches, stomach-aches or nausea (above 5 years old).

#### Employment

We asked respondents between 18 and 60 years old if they had a paid job, either in cash or in goods, what was the employment status (7 categories), if it was full time or part time and since when did they work.

#### Exposure variables

We did not measure the specific effect of each service delivered by the CBR program. Each participant received services tailored to their needs. The program delivered a set of services including physiotherapy, group training, loans, home based education, center-based education, inclusion in school, home based training, community advocacy and disability awareness. In other words, the present study does not measure the impact of the discrete interventions offered by the program but rather the overall impact of the program as a whole and the combination of interventions on the participants. We cannot assess whether it was the physiotherapy or the home-based education that contributed most to the change in outcomes over time, but rather a combination of interventions as deemed necessary and useful for each participant.

#### Covariates

Covariates we kept for PSM calculation included gender (male, female), age (continuous), cause of disability (birth, accident, disease, conflict related, other cause), disability type (physical/locomotor, sensory, intellectual, mental illness & epilepsy, multiple disabilities), ethnicity (Pashtun, Tajik, minority ethnicity), assets owned at baseline in tertile (poorest, 20–80%, highest), region (eastern region, northern region, south eastern region, north eastern region) age of onset of the disability (continuous), household education level (illiterate, some education), working status at baseline (not working, working) and household income at baseline (continuous). Moreover, people were also matched according to the baseline levels of the impact variables to ensure that CBR participants and controls started off at similar levels. We also included community-level variables such as distance to a road (continuous, in kilometres) and its usability for motorized vehicles (usable or not), availability of electricity (available or not), availability or not and distance (continuous, in minutes) to a school and a health center, presence or not of different types of social and/or political groups in the village (international NGO, religious group, political party, village *shura*, education or health *shura*, district development assembly, CBR committee), and exposure to different types of disasters or negative events (natural disaster, attack or other type of crisis such as landslide, drought or inundation) in the last 3 years.

### Statistical methods

We used a quasi-experimental approach that mixed propensity score matching with difference in difference (PSM-DD) to measure the effect of the CBR program. We analyzed the three waves of data collected from treatment and control from surrounding communities, from the onset of the program at baseline until end-line. Yet, some CBR participants left the program before because they did not need the services anymore, some migrated and a few died. Some controls left the study because they did not want to participate anymore, migrated or died. As a result, we only have two interviews for them. The PSM framework used baseline data to find the best possible control match to the persons that received the CBR program. It is based on potential outcomes: The comparison group includes people with disabilities who would have been eligible to receive the CBR program services but who live outside of the program’s catchment areas. The assumption is that the decision on which communities are part of the program is based on observable characteristics. We use a vast set of control variables that are expected to influence both exposure of the program and outcome of interest. We controlled in the analysis for personal (e.g. gender, age, ethnicity, age at disability onset, cause and type of disability, residence, marital status, education level, employment status, assets index, individual and household income) and village (distance to road, electricity, distance to school and the healthcare facility, presence of multiple organizations, negative event outcome in the previous 3 years) characteristics that might have an effect on the impact of the program and were found to also be predictive of exposure to be in the treatment group. We calculated the likelihood of assignment into treatment in the CBR using a probit model based on the same variables used for matching in the PSM. All variables with a few exceptions (household income, presence of a domestic NGO, religious group, village, education or health *shura*) were found to be significant predictors at *p* < .05 level of assignment into the CBR treatment program when compared to reference categories.

Because all those who are eligible within the catchment area were included in the program we initially made the reasonable assumption that participant and control groups have similar characteristics overall [30, 31]. Yet, persons with disabilities in the catchment area could decide not to participate. Therefore, we tested for common support. The balancing tests show that propensity score matching using the nearest neighbor matching (with a 1:1 ratio, and a 0.10 calliper) removed most of the bias between the CBR participant and control groups. We reproduced the matching process using the Gaussian Kernel-based matching algorithm which is characterize by the ability to minimize total distance between matches with its weighting function without significant loss of observations. The Gaussian Kernel estimator was conducted with six separate specifications as sensitivity analysis of findings. These included using the .6 default bandwidth, a medium (.4) and large (.9) bandwidth, as well as 2, 5, and 10% sample trimming, each with the default bandwidth. We finally tested propensity score weighting including a village cluster effect and found consistent results (See Table 1 for the Gaussian Kernel estimator with default bandwidth and the propensity score weighting results).

**Table 1:** Average treatment effect on the treated (ATT) on all outcomes of interest using other matching methods

	Matched sample	Kernel Based Matching (0.6 bandwidth)	95% Confidence interval	Cohen's d Effect size	95% Confidence interval	Matched sample	Propensity score weighting	95% Confidence interval	Cohen's d Effect size	95% Confidence interval
Mobility index	2352	0.13	0.08-018	-0.33	-0.41--0.25	2277	0.12	0.09-0.15	-0.33	-0.41--0.25
Activities of daily living	2190	0.07	0.01-013	-0.24	-0.33--0.16	2045	0.07	0.03-010	-0.24	-0.33--0.16
Communication	2367	0.08	0.02-0.14	-0.35	-0.43--0.26	2218	0.08	0.06-0.12	-0.35	-0.43--0.26
Social participation index	2367	0.14	0.06-0.21	-0.42	-0.51--0.34	2291	0.14	0.10-0.18	-0.42	-0.51--0.34
Emotional well-being	1670	1.18	0.52-1.84	-0.48	-0.58--0.38	1554	1.23	0.85-1.6	-0.48	-0.58--0.38
Employment	1014	0.08	0.02-0.2	-0.22	-0.34--0.10	1007	0.16	0.09-0.22	-0.22	-0.34--0.10

We combined PSM with the difference in difference (DD) approach when different points in time (Y^0^, Y^1^) were captured to account for all unobservable differences that are stable over time therefore eliminating the risk of selection bias even if some unobservable characteristics that lead to the decision on whether to receive the program could not be captured with the variables (X) [24]. We identified the effect of the CBR program by comparing the change in outcomes of interest $\mathrm{E}\left[{\mathrm{Y}}_{\mathrm{t}+1}^1-{\mathrm{Y}}_{\mathrm{t}}^0|\mathrm{D}=1\right]$ of the CBR participants between the period (t) and (t + 1) to the counterfactual $\mathrm{E}\left[{\mathrm{Y}}_{\mathrm{t}+1}^0-{\mathrm{Y}}_{\mathrm{t}}^0|\mathrm{D}=1\right]$ they would have experienced in the absence of the program. This counterfactual is approximated by the change in outcomes of interest $\mathrm{E}\left[{\mathrm{Y}}_{\mathrm{t}+1}^0-{\mathrm{Y}}_{\mathrm{t}}^0|\mathrm{D}=0\right]$ observed in the control group considering the common trend assumption:

$$ \mathrm{E}\left[{\mathrm{Y}}_{\mathrm{t}+1}^0-{\mathrm{Y}}_{\mathrm{t}}^0|\mathrm{D}=1\right]=\mathrm{E}\left[{\mathrm{Y}}_{\mathrm{t}+1}^0-{\mathrm{Y}}_{\mathrm{t}}^0|\mathrm{D}=0\right] $$

We estimated these two counterfactuals by matching treatment and control with key characteristics. To overcome the possible selection bias and the absence of independence between effect and treatment, we assumed conditional independence, i.e. that we observed all the baseline variables (X) that led a person to receive the program. We assumed the existence of common support which implies that only CBR participants that have a probability of being treated also found in any of the controls were included in the analysis. Similarly, controls with an extremely low probability of being treated were not included either. This method has the advantage of not requiring any assumption on whether the program has homogeneous or heterogeneous effects on the model errors and by being non-parametric it can be combined with other methods in order to yield more precise impact measures [32]. We used a logistic regression of the likelihood to receive the program based on baseline variables (X) for the propensity score estimation. After the propensity score, we estimated the average treatment effect on the treated (ATT):

$$ {\mathrm{ATT}}^{\mathrm{D}\mathrm{D}-\mathrm{PSM}}=\frac{1}{{\mathrm{N}}_{{\mathrm{D}}_1}}\sum \limits_{\mathrm{i}\in {\mathrm{D}}_1\cap S}\left[\left({\mathrm{Y}}_{\mathrm{i},\mathrm{t}+1}^1-{\mathrm{Y}}_{\mathrm{i},\mathrm{t}}^0\right)-\sum \limits_{\mathrm{j}\in {\mathrm{D}}_0\cap S}{\mathrm{W}}_{\mathrm{i}\mathrm{j}}\left({\mathrm{Y}}_{\mathrm{j},\mathrm{t}+1}^0-{\mathrm{Y}}_{\mathrm{j},\mathrm{t}}^0\right)\right] $$

Where D_1_ (D_0_) represents the treatment (control) group, w_ij_ the nearest neighbor matching weights, and S the area of common covariate support. PSM makes the standard DD assumption more plausible by forming statistical twin pairs before performing the DD estimator.

PSM-DD allowed for measuring the relative difference in change in outcomes over time between CBR participants and controls, and counteract the fact that not all variables that led to the definition of a catchment area could be considered, and thus addresses the bias generated by this limitation.

We estimated the PSM using only baseline variables to ensure that people were comparable before any interventions took place. We calculated Cohen’s d effect size estimates of the effect of the CBR program. We used STATA 16 for all analyses.

We conducted sensitivity analysis to assess the robustness of our model results in which we modified the socioeconomic and community-level characteristics of covariates. We used household monthly income (continuous) instead of the welfare index based on assets owned at baseline. We used a different age limit for working at baseline (15 years old instead of 18). We used the 13 provinces instead of the four regional offices. We also tested whether our results were sensitive to an alternative specification of the outcome variables. We calculated the model for program effect separately on the different items of the five outcomes: mobility (sit, stand, move inside/outside the home, walk ten steps alone), activities of daily living (can eat, bath, use the restroom and dress without help), communication (speak, understand instructions, express needs, learn new things), social participation (making friends, consulted in family decisions, participate in community activities/ceremonies, feel respected in the community and in the family), emotional wellbeing (being sad, being angry, being worried, having nightmare as dummy variables). We also measured the effect on employment considering working or not as an adult with two different cuts-off at 15 and 18 years old. We found similar significant effects.

## Results

### Participant flow

**Fig. 2:**
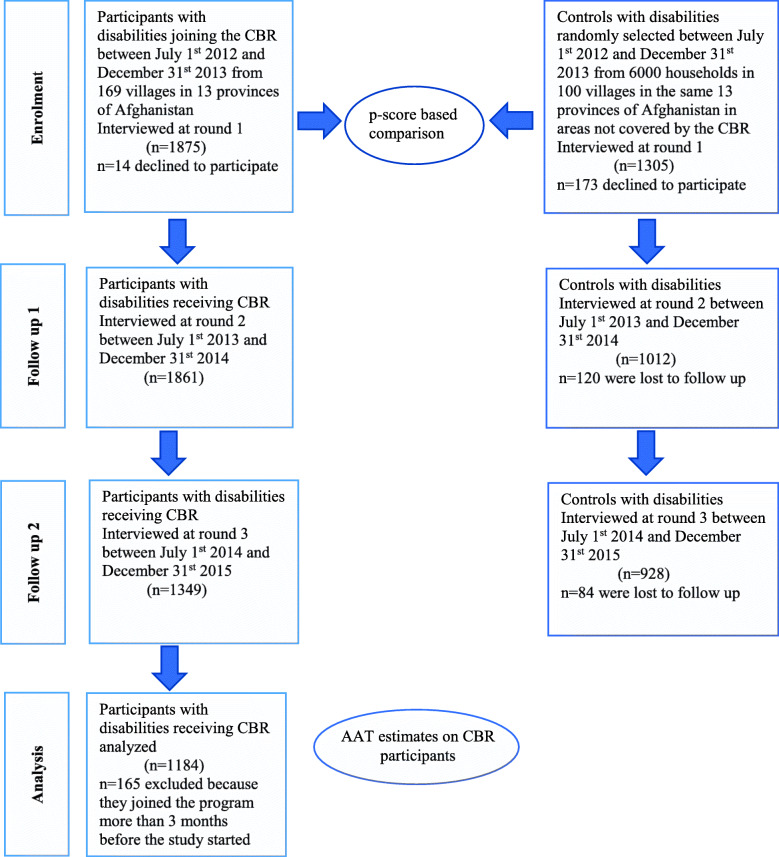
Study participants selection, intervention and follow up process

We interviewed 1875 new CBR participants and 1305 controls at baseline between July 1st 2012 and December 31st 2013. At endline, 1349 CBR participants and 927 controls were interviewed between July 1st 2014 and December 31st 2015 (See Fig. 2). Attrition rate was 28,0 and 28.8% between round 1 and 3 respectively for participants and controls. Reasons for attrition were as follows: death or migration outside of the catchment area of the study as well as no need for further services for participants. Refusal to participate in the study was very minimal among participants (*n* = 14, 0.01%) and higher among controls (refusal at baseline was *n* = 173, 15.3%). Yet, there were few significant differences between respondents and non-respondents with respect to the measured characteristics and we therefore assumed that unobserved data were missing at random [33] (See Table 2).

**Table 2:** Baseline characteristics of treatment and control comparing those with complete exposure and those lost to follow up

	Lost to follow up			Followed up		
	CBR	Control	*P* value	CBR	Control	P value
	n (%)	n (%)		n (%)	n (%)	
Socioeconomic characteristics						
Gender						
Male	308 (60.04)	160 (62.5)	*p* < 0.4332	746 (63.01)	541 (61.76)	*p* < 0.3349
Female	205 (39.96)	96 (37.5)		438 (36.99)	335 (38.24)	
Age (mean, SD)	16.13 (17.83)	35.60 (23.79)	*p* < 0.0001	15.01 (13.79)	29.69 (20.69)	*p* < 0.0001
Disability cause						
Birth	307 (59.84)	66 (25.78)	*p* < 0.0001	702 (59.29)	272 (31.05)	*p* < 0.0001
Accident	48 (9.36)	33 (12.89)		171 (14.44)	131 (14.95)	
Disease	107 (20.86)	61 (23.83)		214 (18.07)	235 (26.83)	
Conflict related	17 (3.31)	22 (8.59)		66 (5.57)	95 (10.84)	
Other cause	34 (6.63)	74 (28.91)		31 (2.62)	143 (16.32)	
Disability type						
Physical/Locomotor	371 (73.9)	108 (57.14)	*p* < 0.0001	824 (69.59)	459 (59.69)	*p* < 0.0001
Sensory	46 (9.16)	42 (22.22)		161 (13.6)	136 (17.69)	
Intellectual	45 (8.96)	10 (5.29)		114 (9.63)	60 (7.8)	
Mental Illness & Epilepsy	2 (0.4)	8 (4.23)		5 (0.42)	37 (4.81)	
Multiple Disabilities	38 (7.57)	21 (11.11)		80 (6.76)	77 (10.01)	
Ethnicity						
Pashtun	143 (27.98)	67 (34.54)	*p* < 0.004	433 (36.57)	331 (38.27)	*p* < 0.1
Tajik	227 (44.42)	108 (55.67)		472 (39.86)	306 (35.38)	
Minority Ethnic	141 (27.59)	19 (9.79)		279 (23.56)	228 (26.36)	
Assets index						
poorest	81 (16.2)	67 (34.54)	*p* < 0.0001	142 (12)	274 (33.5)	*p* < 0.0001
20–80%	333 (66.6)	108 (55.67)		761 (64.33)	424 (51.83)	
Highest	86 (17.2)	19 (9.79)		280 (23.67)	120 (14.67)	
Swedish Committee regional office						
Eastern region	56 (10.92)	76 (29.69)	*p* < 0.0001	215 (18.16)	174 (19.86)	*p* < 0.0001
Northern region	148 (28.85)	94 (36.72)		441 (37.25)	244 (27.85)	
South eastern region	80 (15.59)	35 (13.67)		294 (24.83)	186 (21.23)	
North eastern region	229 (44.64)	51 (19.92)		234 (19.76)	272 (31.05)	
Age of onset of disability (mean, SD)	9.92 (17.55)	21.87 (24.40)	*p* < 0.0001	7.53 (12.74)	15.74 (18.75)	*p* < 0.0001
Some education (ref: illiterate)	49 (9.8)	35 (18.52)	*p* < 0.002	200 (16.91)	142 (18.42)	*p* < 0.390
Employment status (ref. not working)	0 (0)	1 (100)	*p* < 0.002	173 (40.14)	145 (27.62)	*p* < 0.0001
Household income (mean, SD)	6211 (179)	6411 (478)	*p* = 0.696	7987 (808)	6479 (267)	*p* = 0.077
Village characteristics						
Distance to road (kms) (mean, SD)	1.13 (2.49)	2.01 (4.47)	*p* = 0.0036	1.16 (2.18)	2.71 (10.69)	*p* < 0.0001
Road usable by motorized vehicle (ref.: not usable)	496 (96.69)	236 (92.19)	*p* = 0.006	1144 (96.62)	794 (90.64)	*p* < 0.0001
Electricity (ref: no electricity)	456 (88.89)	206 (80.47)	*p* = 0.001	1059 (89.44)	662 (75.57)	*p* < 0.0001
School in the village (ref: no school)	413 (80.51)	204 (79.69)	*p* = 0.788	943 (79.65)	628 (71.69)	*p* < 0.0001
Time to reach school (minutes) (mean, SD)	17.29 (11.72)	17.74 (10.09)	*p* = 0.5784	16.941 (9.71)	19.76 (13.07)	*p* < 0.0001
Healthcare facility in the village (ref: no healthcare facility)	207 (40.35)	131 (51.17)	*p* = 0.004	610 (51.52)	365 (41.67)	*p* < 0.0001
Time to reach healthcare facility (minutes) (mean, SD)	25.66 (20.59)	29.28 (22.48)	*p* = 0.0311	24.001 (17.51)	30.67 (17.51)	*p* < 0.0001
Presence of a self-help group (ref: none)	108 (21.05)	55 (21.48)	*p* = 0.890	270 (22.8)	175 (19.98)	*p* = 0.123
Presence of an International NGO (ref: none)	91 (17.74)	28 (10.94)	*p* = 0.014	303 (25.59)	129 (14.73)	*p* < 0.0001
Presence of a religious group (ref: none)	66 (12.87)	34 (13.28)	*p* = 0.872	208 (17.57)	147 (16.78)	*p* = 0.640
Presence of a political party (ref: none)	66 (12.87)	77 (30.08)	*p* < 0.0001	193 (16.3)	176 (20.09)	*p* = 0.027
Presence of a village *Shurah* (ref.: none)	410 (79.92)	212 (82.81)	*p* = 0.337	967 (81.67)	720 (82.19)	*p* = 0.762
Presence of an education *Shurah* (ref.: none)	228 (44.44)	137 (53.52)	*p* = 0.018	646 (54.56)	416 (47.49)	p = 0.001
Presence of a health *Shurah* (ref.: none)	125 (24.37)	68 (26.56)	*p* = 0.508	368 (31.08)	214 (24.43)	p = 0.001
Presence of a Community development Council (ref.: none)	400 (77.97)	174 (67.97)	*p* = 0,003	898 (75.84)	684 (78.08)	*p* = 0.234
Presence of a business cooperative (ref.: none)	92 (17.93)	6 (2.34)	*p* < 0.0001	216 (18.24)	42 (4.79)	*p* < 0.0001
Presence of a District Development Assembly (ref.: none)	90 (17.54)	83 (32.42)	*p* < 0.0001	227 (19.17)	201 (22.95)	*p* = 0.037
Presence of a CBR committee (ref.: none)	229 (44.64)	12 (4.69)	*p* < 0.0001	670 (56.59)	54 (6.16)	*p* < 0.0001
Village affected by a natural disaster in last 3 years (ref: not affected)	287 (55.95)	141 (55.08)	*p* < 0.0001	580 (48.99)	570 (65.07)	*p* < 0.0001
Village affected by an attack in last 3 years (ref: not affected)	162 (31.58)	46 (17.97)	*p* < 0.0001	356 (30.07)	174 (19.86)	*p* < 0.0001
Village affected by another crisis/disaster in last 3 years (ref: not affected)	380 (74.07)	228 (89.06)	*p* < 0.0001	933 (78.8)	731 (83.45)	*p* < 0.008

### Baseline data

**Fig. 3:**
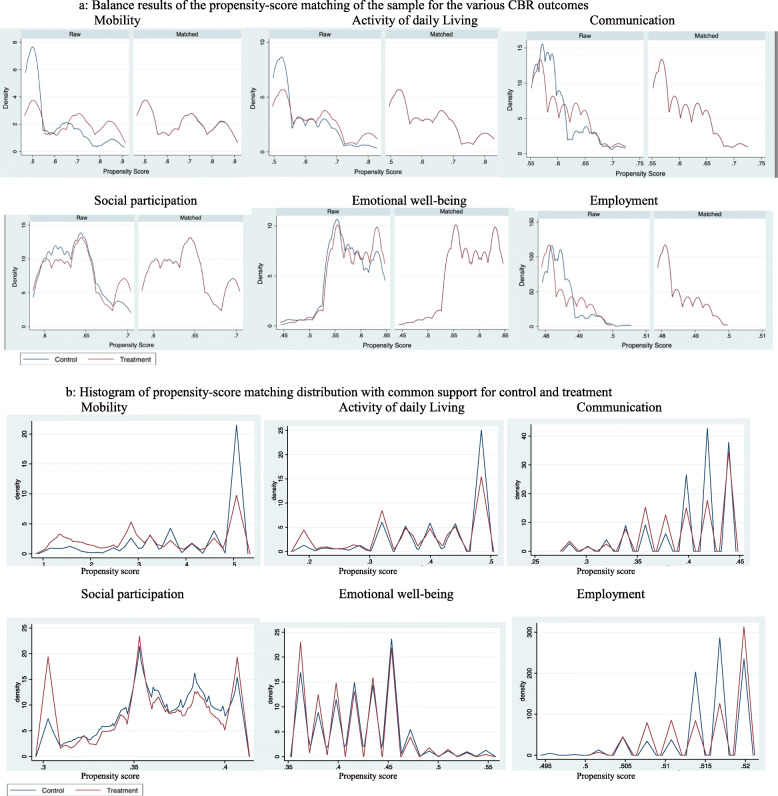
**a** Balance results of the propensity-score matching of the sample for the various CBR outcomes. **b** Histogram of propensity-score matching distribution with common support for control and treatment

Table 3 presents the difference in various characteristics between CBR participants and controls at baseline. Examining the balance before and after PSM (Fig. 3 and Table 2) illustrates that the unmatched CBR and control groups differ substantially in terms of important confounding factors: CBR participants were younger, more often with mobility/locomotor limitations, disabled at birth than controls. They came from families with more material assets and slightly higher average income and were more likely to work than controls. They live in villages more connected to a road, more likely to have electricity, a school or an healthcare facility. Once PSM is calculated, Fig. 3 shows that covariates are well balanced and matching significantly reduces the mean standardised bias for each covariate below the standard threshold of 5% [34].

**Table 3:** Characteristics at baseline of study participants

	Treatment	Control	*P* value
	n (%)	n (%)	
Socioeconomic characteristics			
Gender			
Male	1054 (62.11)	701 (61.93)	*p* = 0.921
Female	643 (37.89)	431 (38.07)	
Age (mean, SD)	15.34 (15.10)	30.85 (21.46	*p* < 0.0001
Disability cause			
Birth	1009 (59.46)	338 (29.86)	*p* < 0.0001
Accident	219 (12.91)	164 (14.49)	
Disease	321 (18.92)	296 (26.15)	
Conflict related	83 (4.89)	117 (10.34)	
Other cause	65 (3.83)	217 (19.17)	
Disability type			
Physical/Locomotor	1195 (70.88)	567 (59.19)	*p* < 0.0001
Sensory	207 (12.28)	178 (18.58)	
Intellectual	159 (9.43)	70 (7.31)	
Mental Illness & epilepsy	7 (0.42)	45 (4.7)	
Multiple Disabilities	118 (7)	98 (10.23)	
Ethnicity			
Pashtun	576 (33.98)	428 (38.32)	*p* = 0.003
Tajik	699 (41.24)	390 (34.91)	
Minority Ethnic	420 (24.78)	299 (26.77)	
Assets index			
poorest	223 (13.25)	341 (33.7)	*p* < 0.0001
20–80%	1094 (65)	532 (52.57)	
Highest	366 (21.75)	139 (13.74)	
Swedish Committee regional office			
Eastern region	271 (15.97)	250 (22.08)	*p* < 0.0001
Northern region	589 (34.71)	338 (29.86)	
South eastern region	374 (22.04)	221 (19.52)	
North eastern region	463 (27.28)	323 (28.53)	
Age of onset of disability (mean, SD)	8.24 (14.38)	16.94 (20.12)	*p* < 0.0001
Education			
Illiterate	1434 (85.2)	783 (81.56)	*p* = 0.114
Some education	249 (14.8)	177 (18.44)	
Employment status (18–65 years old)			
Not working	258 (258)	380 (72.24)	*p* < 0.0001
Working	173 (40.14)	146 (27.76)	
Village characteristics			
Distance to road (kms) (mean, SD)	1.15 (2.28)	2.55 (9.64)	*p* < 0.0001
Road usable by motorized vehicle (ref.: not usable	1640 (96.64)	1030 (90.99)	*p* < 0.0001
Electricity (ref: no electricity)	1515 (89.28)	868 (76.68)	*p* < 0.0001
School in the village (ref: no school)	1356 (79.91)	832 (73.5)	*p* < 0.0001
Time to reach school (minutes) (mean, SD)	17.04 (10.36)	19.31 (12.48)	*p* < 0.0001
Healthcare facility in the village (ref: no healthcare facility)	817 (48.14)	496 (43.82)	*p* = 0.024
Time to reach healthcare facility (minutes) (mean, SD)	24.511 (18.50)	30.35 (24.36)	*p* < 0.0001
Presence of a self-help group (ref: none)	378 (22.27)	230 (20.32)	*p* = 0.215
Presence of an International NGO (ref: none)	394 (23.22)	157 (13.87)	*p* < 0.0001
Presence of a religious group (ref: none)	274 (16.15)	181 (15.99)	*p* = 0.911
Presence of a political party (ref: none)	259 (15.26)	253 (22.35)	*p* < 0.0001
Presence of a village Shurah (ref.: none)	1377 (81.14)	932 (82.33)	*p* = 0.424
Presence of an education Shurah (ref.: none)	874 (51.5)	553 (48.85)	*p* = 0.167
Presence of a health Shurah (ref.: none)	493 (29.05)	282 (24.91)	*p* = 0.016
Presence of a Community development Council (ref.: none)	1298 (76.49)	858 (75.8)	*p* = 0.672
Presence of a business cooperative (ref.: none)	308 (18.15)	48 (4.24)	*p* < 0.0001
Presence of a District Development Assembly (ref.: none)	317 (18.68)	284 (25.09)	*p* < 0.0001
Presence of a CBR committee (ref.: none)	899 (52.98)	66 (5.83)	*p* < 0.0001
Village affected by a natural disaster in last 3 years (ref: not affected)	867 (51.09)	711 (62.81)	*p* < 0.0001
Village affected by an attack in last 3 years (ref: not affected)	518 (30.52)	220 (19.43)	*p* < 0.0001
Village affected by another crisis/disaster in last 3 years (ref: not affected)	1313 (77.37)	959 (84.72)	*p* < 0.0001

### Bivariate analysis of effect of intervention exposure on outcomes

At baseline, CBR participants showed on average higher limitations for all outcomes of interest (See Table 4). A significant higher proportion of CBR participants could not carry-out any of the four mobility activities or had some difficulties compared to controls. Similarly, CBR participants had significantly more difficulties to accomplish any of the activities of daily living. Results for communication comparisons were consistent with limitations of basic activities of daily living and mobility: overall CBR participants had higher rates of complete or partial limitation than controls except for learning new things, where controls have slightly higher limitations. Study results show that CBR participants faced higher barriers to social participation. Interestingly, perception of lack of respect by family was observed to be very low and less frequent in both groups than perception of lack of respect by the community. Yet, participation in family decisions was high as approximately 9% of both CBR participants and controls above age 15 were never consulted. Finally, we found that CBR participants and controls demonstrated very similar and relatively low levels of severe mental distress and anxiety; feeling sometimes worried or angry was even significantly higher among controls.

**Table 4:** Effect of the exposure to the CBR program on outcomes of interest

	Baseline			Endline		
	CBR	Control	*P*-value	CBR	Control	P-value
**Mobility**						
Can you sit by yourself?						
Never/Cannot	206 (12.82)	23 (2.41)	*p* < 0.001	23 (1.81)	13 (1.49)	*p* < 0.001
Sometimes/with difficulty or help	543 (33.79)	119 (12.47)		442 (34.69)	73 (8.35)	
Always/without difficulty	858 (53.39)	812 (85.12)		809 (63.50)	788 (90.16)	
Can you stand by yourself?						
Never/Cannot	406 (25.26)	73 (7.64)	*p* < 0.001	90 (7.05)	45 (5.15)	*p* < 0.001
Sometimes/with difficulty or help	521 (32.42)	186 (19.48)		424 (33.23)	131 (14.99)	
Always/without difficulty	680 (42.31)	696 (72.88)		762 (59.72)	698 (79.86)	
Can you move inside the house by yourself?						
Never/Cannot	460 (28.62)	84 (8.80)	*p* < 0.001	102 (8.00)	44 (5.04)	*p* < 0.001
Sometimes/with difficulty or help	518 (32.23)	254 (26.60)		434 (34.04)	165 (18.90)	
Always/without difficulty	629 (39.14)	617 (64.61)		739 (57.96)	664 (76.06)	
Can you move outside the house by yourself?						
Never/Cannot	448 (30.48)	94 (9.99)	*p* < 0.001	130 (10.98)	52 (6.01)	*p* < 0.001
Sometimes/with difficulty or help	486 (33.06)	326 (34.64)		385 (32.52)	253 (29.25)	
Always/without difficulty	536 (36.46)	521 (55.37)		669 (56.50)	560 (64.74)	
**Activities of daily living**						
Can you feed yourself?						
Never/Cannot	91 (7.13)	30 (3.33)	*p* < 0.001	10 (2.29)	10 (0.96)	*p* < 0.001
Sometimes/with difficulty or help	423 (33.12)	142 (15.74)		348 (33.37)	102 (12.30)	
Always/without difficulty	763 (59.75)	730 (80.93)		685 (65.68)	708 (85.40)	
Can you bathe yourself?						
Never/Cannot	118 (12.90)	60 (7.43)	*p* < 0.001	23 (3.01)	36 (4.90)	*p* < 0.007
Sometimes/with difficulty or help	357 (39.02)	305 (37.79)		209 (27.39)	241 (32.79)	
Always/without difficulty	440 (48.09)	442 (54.77)		531 (69.59)	458 (62.31)	
Can you use the latrine by yourself?						
Never/Cannot	233 (17.14)	53 (5.73)	*p* < 0.001	44 (3.98)	38 (4.47)	*p* < 0.001
Sometimes/with difficulty or help	520 (38.26)	309 (33.41)		375 (33.94)	214 (25.15)	
Always/without difficulty	606 (44.59)	563 (60.86)		686 (62.08)	599 (70.39)	
Can you dress yourself?						
Never/Cannot	203 (15.88)	55 (6.10)	*p* < 0.001	38 (3.64)	33 (3.98)	*p* < 0.001
Sometimes/with difficulty or help	438 (34.27)	256 (28.38)		338 (32.41)	169 (20.39)	
Always/without difficulty	637 (49.84)	591 (65.52)		667 (63.95)	627 (75.63)	
**Communication**						
Can you speak?						
Never/Cannot	345 (23.76)	108 (11.44)	*p* < 0.001	175 (14.77)	79 (9.13)	*p* < 0.001
Sometimes/with difficulty or help	410 (28.24)	132 (13.98)		341 (28.78)	122 (14.1)	
Always/without difficulty	697 (48)	704 (74.58)		669 (56.46)	664 (76.76)	
Can you understand simple instructions?						
Never/Cannot	202 (13.91)	66 (7.01)	*p* < 0.001	40 (3.38)	32 (3.7)	
Sometimes/with difficulty or help	455 (31.34)	163 (17.3)		390 (32.94)	153 (17.69)	
Always/without difficulty	795 (54.75)	713 (75.69)		754 (63.68)	680 (78.61)	
Can you express needs?						
Never/Cannot	267 (18.4)	91 (9.65)	*p* < 0.001	72 (6.08)	39 (4.51)	*p* < 0.001
Sometimes/with difficulty or help	430 (29.63)	158 (16.76)		383 (32.35)	154 (17.8)	
Always/without difficulty	754 (51.96)	694 (73.59)		729 (61.57)	672 (77.69)	
Do you feel confident learning new things?						
Never/Cannot	406(28.57)	349(38.69)	*p* < 0.001	139(13.23)	238(28.64)	*p* < 0.001
Sometimes/with difficulty or help	441(31.03)	279(30.93)		339(32.25)	337(40.55)	
Always/without difficulty	574(40.39)	274(30.38)		573(54.52)	255(30.69)	
**Social participation**						
Can you make friends outside the family?						
Never/Cannot	598(33.98)	162(17.0)	*p* < 0.001	182(15.22)	147(16.84)	*p* < 0.001
Sometimes/with difficulties	491(27.9)	368(38.61)		346(28.93)	317(36.31)	
Always/without difficulties	671(38.12)	423(44.39)		668(55.85)	409(46.85)	
Are you consulted in family decisions?						
Never	58(8.59)	60(9.05)	0.511	19(3.89)	82(13.78)	
Sometimes	212(31.41)	189(28.51)		138(28.28)	157(26.39)	
Always	405(60)	414(62.44)		331(67.83)	356(59.83)	
Can you join in community activities & ceremonies?						
Never	443(27.23)	177(18.75)	*p* < 0.001	94(7.4)	132(15.17)	*p* < 0.001
Sometimes	614(37.74)	498(52.75)		581(45.75)	591(67.93)	
Always	570(35.03)	269(28.5)		595(46.85)	147(16.9)	
Do you feel respected in the community?						
Never	170 (12.4)	78(8.93)	*p* < 0.027	51 (4.94)	69(8.37)	*p* < 0.001
Sometimes	434(31.66)	302(34.59)		29.72(675)	38.59(437)	
Always	767(55.94)	493(56.47)		675(65.34)	437(53.03)	
Do you feel respected in your family?						
Never	39(2.74)	16(1.81)	*p* < 0.001	6(0.58)	5(0.61)	*p* < 0.001
Sometimes	461(32.37)	191(21.66)		342(32.88)	130(15.78)	
Always	924(64.89)	675(76.53)		692(66.54)	689(83.62)	
**Emotional well-being**						
Do you feel sad?						
Always	131(9.22)	63(6.96)	*p* < 0.001	13(1.23)	45(5.42)	*p* < 0.001
Sometimes	656(46.16)	554(61.22)		380(36.05)	565(68.07)	
Never	634(44.62)	288(31.82)		661(62.71)	220(26.51)	
Do you feel angry?						
Always	97(6.83)	56(6.19)	*p* < 0.001	11(1.05)	37(4.46)	*p* < 0.001
Sometimes	680(47.85)	527(58.3)		425(40.59)	608(73.25)	
Never	644(45.32)	321(35.51)		611(58.36)	185(22.29)	
Do you feel worried or distressed?						
Always	120(8.75)	54(6.14)	*p* < 0.001	21(2.01)	37(4.49)	*p* < 0.001
Sometimes	629(45.85)	537(61.09)		393(37.54)	585(71)	
Never	623(45.41)	288(32.76)		633(60.46)	202(24.51)	
Do you have nightmares or bad sleep?						
Always	84(6.1)	61(6.99)	*p* < 0.188	10(0.96)	25(3.03)	*p* < 0.001
Sometimes	537(38.97)	308(35.28)		376(35.95)	380(46.12)	
Never	757(54.93)	504(57.73)		660(63.1)	419(50.85)	
Do you have headaches, stomachaches or nausea?						
Always	42(3.05)	35(4.01)	*p* < 0.433	11(1.05)	17(2.06)	*p* < 0.001
Sometimes	707(51.34)	451(51.72)		469(44.84)	556(67.48)	
Never	628(45.61)	386(44.27)		566(54.11)	251(30.46)	
**Can you work** (only 18 to 65 years old)?						
Working **(**ref: not working)	173 (40.14)	146 (27.76)	*p* < 0.001	208 (47.82)	150 (25.08)	*p* < 0.001

At end-line, all bivariate analysis showed that the difference between CBR participants and controls remained statistically significant on all variables of interest. The proportion of CBR participants who had severe activity limitations or functioning difficulties in terms of mobility, activity of daily living, communication and social participation or a low level of emotional wellbeing at baseline was smaller at endline. The proportion of CBR participants with severe activity limitations or functioning difficulties or low level of emotional wellbeing at end-line were similar or lower than controls.

### Measuring the effect of the CBR program using propensity score matching with difference in difference analysis

**Fig. 4:**
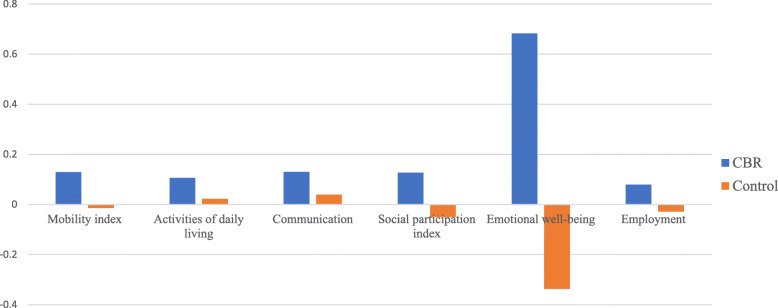
Impact of the CBR program on outcomes of interest using PSM-DID

We use PSM-DD to assess the CBR program impact on the various outcomes of interest. Findings reported in Fig. 4 and Table 5 show a positive impact of the CBR program on all outcomes of interest. Table 5 assesses the ATT including Cohen’s d effect size. The first graph in Fig. 4 presents the change over time in the mobility index for both CBR participants and controls. While the controls saw their mobility worsen slightly by 1.4%, CBR participants’ mobility index improved by 13%. The 14.4 percentage point difference between both groups is statistically significant and the effect size (ES) is of − 0.36 (95% Confidence interval − 0.44– − 0.29), regarded in the literature to be between small and medium size [35, 36]. The second graph shows that both CBR participants and controls improved their capacity to carry out activities of daily living. However, CBR program participants improved by 8.4% more than controls with an ES of − 0.26 (95%CI -0.35– -0.18). Similarly, as shown in the third graph, CBR participants improved their communication abilities by 9.1% more than controls (ES = -0.38, 95%CI -0.46– -0.30). The fourth and fifth graphs show a difference in improvement in social participation skills and emotional wellbeing between CBR participants and controls of 17.8% (ES = -0.45, 95%CI -0.53– -.036) and 10.2% (ES = -0.48, 95%CI -0.58 – − 0.38) respectively. Controls even saw their mobility, social participation skills and emotional wellbeing decrease during the 3 years period of intervention. The last graph in Fig. 4 shows that the CBR program promoted access to employment for participants —by 7.5% on average during the three-year period— while controls underwent a reduction in employment rate during the same period of 4.4% but with a relatively small effect size (ES = -0.21, 95%CI -0.33– − 0.10), in fact the smaller of all program effect.

**Table 5:** Average treatment effect on the treated (ATT) on all outcomes of interest

	Matched sample	ATT	95% Confidence interval	Cohen’s d Effect size	95% Confidence interval
Mobility index	2352	0.14	0.10–0.18	-0.36	−0.44--.29
Activities of daily living	2190	0.08	0.03–0.13	−0.26	−0.35--0.18
Communication	2367	0.09	0.01–0.17	−0.38	−0.46--0.3
Social participation index	2367	0.18	0.12–0.23	−0.45	−0.53--0.36
Emotional well-being	1985	1.02	0.04–2.00	−0.48	− 0.58--0.38
Employment	1014	0.11	0.06–0.18	−0.21	−0.33--0.10

## Discussion

Our study shows that the CBR program had a significant positive impact on several outcomes of interest promoted by the WHO, namely individual mobility, activities of daily living, communication skills, emotional wellbeing, social participation and employment. Our findings suggest that CBR programs can improve livelihoods and wellbeing of persons with disabilities in LMICs [8, 13, 20, 37]. Yet, existing studies present multiple limitations —small sample size, inadequate sample methodology, mostly observational or qualitative approaches, recall bias in the only existing quasi experiment and lack of accounting for confounding factors in regression analysis [8, 38]. Our study follows and interviews a large group of CBR participants and a random group of controls multiple times over a period of 3 years.

Our study is unique in that: (i) it combines a measure of outcomes associated with one of the five components of the CBR matrix —health, education, livelihood, social and empowerment; (ii) it addresses the issue of CBR program sustainability by looking at data from three rounds of interviewing the same CBR cohort of participants and controls with disabilities, showing the lasting effect of the rehabilitation interventions; and (iii) it is one of so far only nine identified studies looking at CBR effectiveness carried out in a low income country; the only one in a conflict context. This paucity of existing evidence makes studies like the present one essential to legitimize the promotion of CBR while shedding light on the conditions of its success. This is of utmost importance in LMICs as CBR has been, especially in crisis and emergency contexts, the only set of services available to persons with disabilities, a particularly disadvantaged group in such contexts [39, 40].

### Interpretation

The CBR program has a differential impact on the various outcomes indicating that improvement is not uniform but depends on the effectiveness of the program in improving the condition of persons with disabilities within a given domain of the CBR matrix rather than the capacity for change within each domain. The highest positive impact is observed on emotional wellbeing. After 3 years on average in the program, the estimated effect size is − 0.48 The gap between treated and controls is particularly salient because the emotional wellbeing of controls without any dedicated support decreased by 34% during the period. While we observed such an increase in anxiety and distress among controls during the three years of observation, CBR participants showed a continuous improvement. Anxiety and distress are a major ill in conflict affected contexts such as Afghanistan. Literature traditionally attributed mental distress to the violence itself [41–45] including sexual violence and intimate partner violence [46–49]. Increasingly, scholars have also shown that daily stressors play an important role in common and more severe mental disorders, particularly among vulnerable groups. Conflict exacerbates economic deprivation adding an important source of mental distress, particularly for those at risk of being economically and socially excluded [50–52]. Literature has shown that persons with disabilities are also particularly at risk of mental distress in conflict settings because of poverty, unemployment but also stigma associated with disability [53, 54].

Another important effect of the program was observed on social participation which encompasses important dimensions of social life. The social participation index reveals a person’s sense of self-worth and placement within their family and community, and also indicates to what extent disability isolates a person from family and community daily life. Effectively changing community attitudes resulting in reduced marginalization and discrimination of persons with disabilities is a major milestone difficult to achieve because of deeply entrenched negative beliefs [54, 55]. Early CBR literature showed that it was easier to restore individuals’ functions than to change contextual factors such as attitudes towards disability [56]. Yet, it is a significative achievement, one that embodies the philosophical underpinning of the CBR principles as defined by the World Health Organization: a bottom-up multipronged set of interventions relying on ownership and empowerment of persons with disabilities [2]. If this outcome reflects genuine social participation and reduction in prejudice, such achievement also contributes to the principle of “full inclusion and participation in all aspects of life” promoted by the article 26 of the UNCRPD. The fact that controls saw their social involvement in their community and family slightly reduced by 5.1% suggests that cultural norms and beliefs foster social isolation that only an external intervention promoting awareness and advocacy can progressively and partially remedy [8, 53, 57].

The significant effect on activities of daily living, communication and mobility demonstrate that a CBR program implemented with limited resources, family support in remote areas prone to violence and disruption associated with conflict, can be effective in providing rehabilitation services through a combination of low cost home-based services such as physiotherapy and access to free orthopedic workshops [58]. Literature has shown that caregivers’ involvement is key in effectively improving participants with disabilities’ functioning outcomes [59]. However, by improving the autonomy of persons with disabilities through rehabilitation processes involving family caregivers, the CBR program reduces the burden that otherwise rest exclusively on them in most LMICs in absence of rehabilitation services [60]: The limited amount of improvement made over the same period of time among controls with disabilities tends to suggest the restricted access to such services in the mainstream healthcare system of Afghanistan.

The program also had a significant marginal effect of 12% on employment of adult participants between 18 and 60 years of age compared to control with disabilities of the same age group. This is a major finding considering there are few interventions and even fewer studies showing the impact of CBR programs tackling the livelihood component of the CBR matrix and supporting employment in LMICs [8]. Such gap in intervention makes escaping poverty highly elusive for persons with disabilities. Overcoming barriers to employment of persons with disabilities is a considerable economic challenge overall. An already 16 years old study estimated at the time that the global gross domestic product loss due to disability to be between $1.71 trillion to $2.23 trillion annually [16, 61]. Furthermore, lack of livelihoods intervention undermines resilience and self-esteem of persons with disabilities, particularly men. In Afghanistan, persons with disabilities have been shown to face lower employment and higher multidimensional poverty compared to non-disabled people particularly for young men [62]. Yet, men are traditionally expected to be breadwinners and look after the needs of the family [63, 64]: For persons with disabilities, the CBR intervention helps secure a job, an essential step towards building a sense of wellbeing resulting from meeting their social obligations and obtaining recognition within the community [65].

The fact that the program was effective in tackling emotional wellbeing, social participation and access to jobs shows that it had an effect on prejudice and discrimination that persons with disabilities face in their community, often the most complex and arduous barrier to overcome [20]. In particular, there is abundant literature exploring the impact of stigma on emotional wellbeing [66, 67]. One of the central goals of CBR programs in general is to challenge stigma of rural communities towards disability in order to promote people with disabilities’ community participation and inclusion [68]. Rehabilitation remains incomplete unless it addresses stigma and prejudice towards people with disabilities who frequently cannot envision the future and have no prospect of social inclusion. It is well established that community acceptance is associated with higher emotional wellbeing and self-esteem but we lack evidence of effective stigma-reduction interventions particularly in low income countries [69]. Similarly to the Swedish Committee for Afghanistan CBR program, another program in Afghanistan promoted by HealthNet TPO also relied on including people with mental disorders and the community in advocacy [70].

### Limitations

Our study presents some limitations. First, there was a risk of self-selection in the CBR program. Elderly people and people with associated disabilities were significantly less likely to participate in the program [58]. There was a risk of selection on non-observable variables as well that we tried to minimize by including a large set of control variables in the propensity score calculation and by using difference in difference as an additional methodology to estimate an unbiased impact. We ran different PSM-DD matching algorithm and found similar impact of the CBR program. We also conducted robustness checks by changing the set of baseline variables (X) for the propensity score estimation and our results hold. Second, there is a risk of information bias as data collection was carried out by CBR workers under the supervision of the investigators and research officers because of security issues. This might have introduced a social desirability bias among CBR workers willing to show a good image of their program. Thorough training*,* careful and ongoing supervision in the field and after data collection, consistency checks as well as random re-interviews of participants has made very unlikely that this bias could change our findings. The items in the emotional index did not have a time limit. We asked respondents to assess if currently they felt sad, *angry, worried, or had nightmare* without indicating in the last seven days for instance. There has been some contamination of the program as few activities benefited controls. In particular, persons with disabilities from villages outside the catchment area would seek assistive devices (orthotics, orthopedics, crutches, wheelchairs) in urban workshops –as well as health rehabilitation services such as physiotherapy in health clinics– run by Swedish Committee for Afghanistan. We found that less than 2% received any kind of service, mostly assistive devices,except for physiotherapy received by 10% of controls (data not shown). Contamination of the program to the benefit of controls means that our findings are probably slightly underestimated. Finally, the selection of catchment areas was not random. However, baseline information shows that CBR participants started worse off than their counterparts, reducing the chance that observed impacts were due to initial differences favoring CBR participants.

## Conclusions

Our results have important implications regarding the capacity of CBR to complement existing government initiatives in addressing the issues related to rehabilitation, autonomy social and economic inclusion of persons with disabilities in a conflict or disaster context and beyond in other LMICs. Better mobility activities of daily living and communication skills are indicators of a CBR program that offers effective rehabilitation services. Most importantly, improvement in emotional wellbeing, social participation and even access to jobs show that the CBR program was also effective in advocating for the rights of persons with disabilities and promoting awareness of disability in the community to fight stigma [20].

The lack of standardization in the CBR program we examined did not allow for identification of specific processes that were most successful in promoting social inclusion. Future research should explore such processes and mechanisms that lead to effective CBR interventions towards persons with disabilities. Process evaluation based on mixed methods to dwell deeper into the process of change using implementation research would help better understand how the program work to be able to standardize the intervention and translate it in a different context.

## Data Availability

Data collected for the study, including deidentified individual participant data and a data dictionary defining each field in the set will be made available to others at publication. The data will be made available in the DRYAD repository at https://datadryad.org/stash with investigator support, after approval of a proposal, with a signed data access agreement.

## Appendix

### Note for the control selection process

We used a random number generator to select a first village to include in the sample from the complete list of villages in each region. The subsequent villages were then selected from the list at the sample interval. This process was repeated for all 13 provinces in the study to compile the full list of 100 control villages. 60 households were randomly selected in each village for a total of 6000 households in the sample. In the social center of the village, typically a mosque or an open square, a child was asked to spin a spinner to determine a direction in the village for the selection of the 60 households to interview. The child was then asked to select randomly a number between 1 and 60 from a small bag to identify the first selected household. Following households were then selected using the nearest front door method: Household number two would be the closest door from household number one’s door. A household was defined as a unit that shared a kitchen, an income and occupied the same flat, house or compound.

## Abbreviations

CBR: Community based rehabilitation
LMIC: Low and middle income country
DSQ34: 34 Items disability screening questionnaire
SCA: Swedish committee for afghanistan
WHO: World health organization
UNCRPD: United Nations convention on the rights of persons with disabilities

## References

[1] United Nations (2006). Convention on the rights of persons with disabilities.

[2] World Health Organization (2010). Community-based rehabilitation: CBR guidelines.

[3] ILO, UNESCO (2004). WHO. Community based rehabilitation: a strategy for rehabilitation, equalization of opportunities, poverty reduction and social inclusion of people with disabilities. Joint position paper.

[4] DFID. Disability, poverty and development. 2000.

[5] Eldar R (2000). Integrated institution - community rehabilitation in developed countries: a proposal. Disabil Rehabil.

[6] Helander E, United Nations Development Programme (1993). Interregional Programme for disabled P. prejudice and dignity: an introduction to community-based rehabilitation.

[7] Mitchell R (1999). The research base of community-based rehabilitation. Disabil Rehabil.

[8] Mauro V, Biggeri M, Grilli L (2015). Does community-based rehabilitation enhance the multidimensional well-being of deprived persons with disabilities? a multilevel impact evaluation. World Dev.

[9] Cornielje H (2009). The role and position of disabled People’s Organisations in community based rehabilitation: balancing between dividing lines. Asia Pac Disabi Rehabil J.

[10] Sharma M (2007). Community participation in community-based rehabilitation programmes. Asia Pac Disabi Rehabil J.

[11] Turmusani M, Vreede A, Wirz SL (2002). Some ethical issues in community-based rehabilitation initiatives in developing countries. Disabil Rehabil.

[12] Madden RH, Lukersmith S, Millington MJ, Scarf C, Fortune N, Hartley S, Llewellyn G (2016). Participatory monitoring of community-based rehabilitation and other disability-inclusive development programmes: the development of a manual and menu. Disabil CBR Inclusive Dev.

[13] Chappell P, Johannsmeier C (2009). The impact of community based rehabilitation as implemented by community rehabilitation facilitators on people with disabilities, their families and communities within South Africa. Disabil Rehabil.

[14] Kuyini AB, Alhassan A-RK, Mahama FK (2011). The Ghana community-based rehabilitation program for people with disabilities: what happened at the end of donor support?. J Soc Work Disabil Rehabil.

[15] Iemmi V, Blanchet K, Gibson LJ, Kumar KS, Rath S, Hartley S, Murthy GVS, Patel V, Weber J, Kuper H (2016). Community-based rehabilitation for people with physical and mental disabilities in low- and middle-income countries: a systematic review and meta-analysis. J Dev Effectiveness.

[16] Saran A, White H, Kuper H. Evidence and gap map of studies assessing the effectiveness of interventions for people with disabilities in low-and middle-income countries. Campbell Syst Rev. 2020;16(1):e1070. 10.1002/cl2.1070 PMC8356326 37131970

[17] Mitchell RA, Zhou D, Lu Y, Watts G (1993). Community-based rehabilitation: does it change community attitudes towards people with disability?. Disabil Rehabil.

[18] Hartley S, Finkenflugel H, Kuipers P, Thomas M (2009). Community-based rehabilitation: opportunity and challenge. Lancet.

[19] Mijnarends D, Pham D, Swaans K, Van Brakel W, Wright P (2011). Sustainability criteria for CBR programmes–two case studies of provincial programmes in Vietnam. Disabil CBR Inclusive Devel.

[20] Biggeri M, Deepak S, Mauro V, Trani JF, Kumar J, Ramasamy P (2014). Do community-based rehabilitation programmes promote the participation of persons with disabilities? A case control study from Mandya District, in India. Disabil Rehabil.

[21] Alavi Y, Kuper H. Evaluating the impact of rehabilitation in the lives of people with disabilities and their families in low and middle income countries: a review of tools. UK: London School of Hygiene & Tropical Medicine; 2010. p. 100.

[22] Cornielje H, Velema JP, Finkenflügel H (2008). Community based rehabilitation programmes: monitoring and evaluation in order to measure results. Lepr Rev.

[23] Velema JP, Ebenso B, Fuzikawa PL (2008). Evidence for the effectiveness of rehabilitation-in-the-community programmes. Lepr Rev.

[24] Heckman JJ, Ichimura H, Todd PE (1997). Matching as an econometric evaluation estimator: evidence from evaluating a job training Programme. Rev Econ Stud.

[25] Rubin DB (2005). Causal inference using potential outcomes: design, modeling, decisions. J Am Stat Assoc.

[26] Guo S, Fraser MW. Propensity score analysis: statistical methods and applications. Thousand Oaks: SAGE publications; 2014.

[27] World Health Organisation (1989). Training in the community for people with disabilities: guide for local supervisors.

[28] Trani JF, Babulal GM, Bakhshi P. Development and validation of the 34-item disability screening questionnaire (DSQ-34) for use in low and middle income countries epidemiological and development surveys. Plos One. 2015;10(12). 10.1371/journal.pone.0143610 PMC4667846 26630668

[29] DeMaio T, Rothgeb J, Schwarz N, Sudman S (1996). Cognitive interviewing techniques: in the lab and in the field. Answering Questions Methodology for Determining Cognitive and Communicative Processes in Survey Research.

[30] Rosenbaum PR, Rubin DB (1983). The central role of the propensity score in observational studies for causal effects. Biometrika..

[31] Rubin DB (1974). Estimating causal effects of treatments in randomized and nonrandomized studies. J Educ Psychol.

[32] Blundell R, Costa DM (2002). Alternative approaches to evaluation in empirical microeconomics. Port Econ J.

[33] Little RJ, Rubin DB. Statistical analysis with missing data. Thousand Oaks: John Wiley & Sons; 2014.

[34] Caliendo M, Kopeinig S (2008). Some practical guidance for the implementation of propensity score matching. J Econ Surv.

[35] Cohen J. Statistical power analysis for the behavioral sciences. Thousand Oaks: Academic press; 2013. 10.4324/9780203771587.

[36] Sawilowsky SS (2009). New effect size rules of thumb. J Mod Appl Stat Methods.

[37] Mauro V, Biggeri M, Deepak S, Trani JF (2014). The effectiveness of community-based rehabilitation programmes: an impact evaluation of a quasi-randomised trial. J Epidemiol Community Health.

[38] Patel S, Alavi Y, Lindfield R, Kuper H (2013). The impact of rehabilitative services in the lives of adults and children with disabilities, in low-income and middle-income countries: an assessment of the quality of the evidence. Disabil Rehabil.

[39] dos Santos-Zingale M, McColl MA (2006). Disability and participation in post-conflict situations: the case of Sierra Leone. Disabil Soc.

[40] Kett M, van Ommeren M (2009). Disability, conflict, and emergencies. Lancet.

[41] Miller K, Rasmussen A (2010). War exposure, daily stressors, and mental health in conflict and post-conflict settings: bridging the divide between trauma-focused and psychosocial frameworks. Soc Sci Med.

[42] Reed RV, Fazel M, Jones L, Panter-Brick C, Stein A (2012). Mental health of displaced and refugee children resettled in low-income and middle-income countries: risk and protective factors. Lancet..

[43] Steel Z, Chey T, Silove D, Marnane C, Bryant RA, Van Ommeren M (2009). Association of torture and other potentially traumatic events with mental health outcomes among populations exposed to mass conflict and displacement: a systematic review and meta-analysis. Jama..

[44] Llosa AE, Casas G, Thomas H, Mairal A, Grais RF, Moro M-R (2012). Short and longer-term psychological consequences of operation cast Lead: documentation from a mental health program in the Gaza strip. Confl Heal.

[45] Londoño A, Romero P, Casas G (2012). The association between armed conflict, violence and mental health: a cross sectional study comparing two populations in Cundinamarca department, Colombia. Conflict Health.

[46] Tol WA, Stavrou V, Greene MC, Mergenthaler C, Van Ommeren M, García MC. Sexual and gender-based violence in areas of armed conflict: a systematic review of mental health and psychosocial support interventions. Conflict Health. 2013;7(1). 10.1186/1752-1505-7-16 PMC3750365 23915821

[47] Kiss L, Quinlan-Davidson M, Pasquero L, Tejero PO, Hogg C, Theis J, et al. Male and LGBT survivors of sexual violence in conflict situations: A realist review of health interventions in low-and middle-income countries. Conflict Health. 2020;14(1). 10.1186/s13031-020-0254-5 PMC7045597 32127913

[48] Horn R, Puffer ES, Roesch E, Lehmann H (2014). Women’s perceptions of effects of war on intimate partner violence and gender roles in two post-conflict west African countries: consequences and unexpected opportunities. Confl Heal.

[49] Murphy M, Ellsberg M, Contreras-Urbina M (2020). Nowhere to go: disclosure and help-seeking behaviors for survivors of violence against women and girls in South Sudan. Confl Heal.

[50] Miller K, Omidian P, Rasmussen A, Yaqubi A, Daudzi H (2008). Daily stressors, war experiences, and mental health in Afghanistan. Transcult Psychiatry.

[51] Betancourt T, Agnew-Blais J, Gilman S, Ellis B (2010). Past horrors, present struggles: the role of stigma in the association between war experiences and psychosocial adjustment among former child soldiers in Sierra Leone. Soc Sci Med.

[52] Panter-Brick C, Eggerman M, Gonzalez V, Safdar S (2009). Violence, suffering, and mental health in Afghanistan: a school-based survey. Lancet.

[53] Trani JF, Bakhshi P (2013). Vulnerability and mental health in Afghanistan: looking beyond war exposure. Transcult Psychiatry..

[54] Trani JF, Ballard E, Pena J (2016). Stigma of persons with disabilities in Afghanistan: examining the pathways from stereotyping to mental distress. Soc Sci Med.

[55] Cerveau T, Trani JF (2011). Deconstructing myths; facing reality. Understanding social representations of disability in Afghanistan. Development efforts in Afghanistan: Is there a will and a way? The case of disability and vulnerability. Ethique economique.

[56] Thomas M, Thomas MJ (1999). A discussion on the shifts and changes in community based rehabilitation in the last decade. Neurorehabil Neural Repair.

[57] Trani JF, Bakhshi P, Trani JF (2011). Profiling and understanding people with disabilities in Afghanistan. Development effort in Afghanistan: Is there a will and a way? The case of disability and vulnerability. Ethique economique.

[58] Trani JF, Vasquez-Escalon J, Bakhshi P (2017). Home based intervention disability program impact evaluation study. Final report.

[59] Asher L, Hanlon C, Birhane R, Habtamu A, Eaton J, Weiss HA, et al. Community-based rehabilitation intervention for people with schizophrenia in Ethiopia (RISE): A 12 month mixed methods pilot study. BMC Psychiatry. 2018;18(1). 10.1186/s12888-018-1818-4 PMC6091097 30075715

[60] Evans PJ, Zinkin P, Harpham T, Chaudury G (2001). Evaluation of community-based rehabilitation for disabled persons in developing countries. Soc Sci Med.

[61] Metts R, Mondiale B. Disability and Development, background paper for the World Bank. World Bank Washington DC Retrieved from http://siteresources worldbank org/DISABILITY/Resources/280658–1172606907476/mettsBGpaper pdf. 2004.

[62] Trani J-F, Kuhlberg J, Cannings T, Chakkal D (2016). Multidimensional poverty in Afghanistan: who are the poorest of the poor?. Oxf Dev Stud.

[63] Dupree N, Trani JF (2011). The historical and cultural context of disability in Afghanistan. Development effort in Afghanistan: Is there a will and a way? The case of disability and vulnerability. Ethique economique.

[64] Centlivres P, Centlivres-Demont M (1988). Et si on parlait de l’Afghanistan?.

[65] Trani JF, Bakhshi P, Rolland C (2011). Capabilities, perception of well-being and development effort: some evidence from Afghanistan. Oxf Dev Stud.

[66] Hatzenbuehler ML, Phelan JC, Link BG (2013). Stigma as a fundamental cause of population health inequalities. Am J Public Health.

[67] Shtayermman O (2009). An exploratory study of the stigma associated with a diagnosis of asperger's syndrome: the mental health impact on the adolescents and young adults diagnosed with a disability with a social nature. J Hum Behav Soc Environ.

[68] World Health Organization, World Bank (2011). World report on disability.

[69] Li L, Guan J, Liang LJ, Lin C, Wu Z (2013). Popular opinion leader intervention for HIV stigma reduction in health care settings. AIDS Educ Prev.

[70] Ventevogel P, van de Put W, Faiz H, van Mierlo B, Siddiqi M, Komproe IH. Improving access to mental health care and psychosocial support within a fragile context: a case study from Afghanistan. Plos Med. 2012;9(5). 10.1371/journal.pmed.1001225 PMC3362640 22666183

